# Enhancing Tribo-Mechanical and Corrosion Properties of ADC 12 Alloy Composites through Marble Dust Reinforcement by Squeeze Casting Technique

**DOI:** 10.3390/ma16196374

**Published:** 2023-09-24

**Authors:** Palanivendhan Murugadoss, Chandradass Jeyaseelan

**Affiliations:** Center for Automotive Materials, Department of Automobile Engineering, College of Engineering and Technology, SRM Institute of Science and Technology, Kattankulathur, Chengalpattu 603203, Tamil Nadu, India

**Keywords:** Aluminum Metal Matrix Composite (AMMC), ADC 12 alloy, marble dust, linear reciprocating tribometer, squeeze casting

## Abstract

This research intends to enhance the tribo-mechanical and corrosion properties of ADC 12 alloys by incorporating marble dust (MD) as a reinforcing element. Composites with varied MD concentrations (0–10 wt%) were fabricated using a squeeze casting process, addressing the limitations of conventional casting techniques. The microstructural analysis confirmed homogeneous MD dispersion within the ADC 12 matrix, facilitating an effective load transfer and solid interfacial bonding. As MD content increased, the experimental density decreased, while porosity increased from 1.22% to 3.97%. Remarkably, adding 4 wt% MD yielded a 20.41%, 17.63%, and 15.75% enhancement in hardness, tensile, and compression strength compared to the as-cast ADC 12. Incorporating MD particles facilitated Orowan strengthening and Hall–Petch strengthening mechanisms, contributing to the observed improvements. The wear rate was reduced by 18.33% with MD content, showing a 17.57% corrosion reduction at 72 h. These outcomes establish the synergistic benefits of ADC 12 squeeze casting with MD reinforcement, delivering superior tribo-mechanical and corrosion properties.

## 1. Introduction

In recent years, pursuing advanced materials with improved mechanical, tribological, and corrosion-resistant properties has gained significant attention in engineering and industrial applications. Aluminum alloys, known for their lightweight and favorable mechanical characteristics, have been extensively used in various sectors, particularly the automotive industry [1]. Among these alloys, ADC 12 stands out as a well-regarded aluminum–silicon (Al-Si) alloy, valued for its exceptional castability, corrosion resistance, durability, and mechanical properties [2]. This material has a range of uses, notably manufacturing automotive components such as cylinder head covers, engine mounts, valves, and so on. However, to further enhance its performance and address specific application requirements, novel approaches are sought that can overcome the limitations of conventional reinforcement materials and casting techniques.

Given that ADC 12 alloys are commonly reinforced with a wide array of ceramic particles, such as Al_2_O_3_, ZrO_2_, SiC, B_4_C, CNTs, TiB_2_, TiC, MgO, and fibers/whiskers, to enhance the mechanical, thermal, and tribological properties of the composite [3]. However, these conventional reinforcing elements pose challenges such as high cost and limited reinforcing elements availability [4]. In addition, obtaining and processing these ceramic reinforcements are energy-intensive, leading to higher carbon emissions and environmental impact [5]. Furthermore, incorporating some conventional ceramic particles often results in poor interfacial bonding with the ADC 12 matrix, affecting the composite’s mechanical properties [6]. The potential toxicity and health hazards associated with certain ceramic materials, such as B_4_C, CNTs, and SiC nanoparticles, raise concerns about safe handling and disposal challenges [7]. Considering these technical challenges and the pressing need for sustainable materials, exploring alternative reinforcement elements such as industrial wastes becomes crucial [8]. Industrial-based ceramic waste offers several advantages, including cost-effectiveness, easy availability, and eco-friendliness [9], making it a viable option for improving the mechanical and tribological properties of ADC 12 alloys without exacerbating concerns related to resource consumption and environmental impact. This also contributes to achieving sustainable development goals (SDG-12) for responsible consumption and production [10].

Besides the challenges associated with reinforcement, the conventional manufacturing process of ADC 12 alloy also presents certain limitations. Conventional casting techniques for producing ADC 12 alloys include gravity casting, stir casting, centrifugal casting, and die-casting [11]. Gravity casting often produces end-products with porosities and inhomogeneous particle distribution due to inadequate stirring during alloy solidification [12]. The capability of attaining homogeneous particle distribution is the challenge for Centrifugal casting, especially in complex geometries, affecting the mechanical properties of the composites [13]. Attaining uniform distribution and strong interfacial bonding between the matrix and reinforcements is challenging with Stir casting [14]. Die-casting is generally prone to thermal stresses and is limited by the complexity of molds for intricate shapes [15]. In contrast, the two-step stir-cum-squeeze casting approach addresses these challenges. The pre-stirring step in the process ensures better particle dispersion and homogeneous bonding. Subsequent squeeze casting improves density, reduces porosities, and enhances the overall quality of the ADC 12 composites [16].

The literature on ADC 12 alloys and conventional ceramic reinforcements shows a notable lack of comprehensive studies focusing on alternative materials and manufacturing techniques to address both materials and manufacturing challenges. Yashpal et al. [17], in their review, found that, though some studies have explored the incorporation of various ceramic reinforcements, such as Al_2_O_3_, SiC, and CNTs, the high cost, limited availability, and potential toxicity of these reinforcements remain significant concerns for sustainable and eco-friendly composite fabrication. Furthermore, Mussatto et al. [18] reviewed and reported that while conventional casting techniques have been extensively employed, there is limited research on the utilization of a two-step stir-cum-squeeze casting approach, which has the potential to overcome the shortcomings of traditional casting methods, leading to improved mechanical properties and reduced porosity.

An extensive literature survey revealed that industrial MD waste emerges as an attractive alternative reinforcement material due to its abundance as a byproduct in marble processing industries. Specifically, concerning India, the country stands as the world’s third-largest producer of marble, with a substantial share of ~10% of global marble powder extraction originating from quarries within the country. With an annual production increase of 8.8% since the turn of the century, generating significant amounts of MD during the cutting and treatment processes has raised concerns regarding waste disposal practices and associated environmental impact [19]. Utilizing MD addresses the challenges of high cost and limited availability associated with conventional ceramic reinforcements and aligns with sustainable practices by repurposing waste material, contributing to responsible consumption and production (SDG 12) [20]. In this context, marble dust (MD), an industrial waste product, emerges as an intriguing candidate due to its composition, availability, and potential positive effects on the properties of the resulting composites. MD, predominantly composed of calcium carbonate (CaCO_3_) and other trace elements, offers the opportunity to impart desirable attributes to the matrix alloy [21].

From the manufacturing standpoint, the two-step stir-cum-squeeze casting technique offers a promising solution to enhance the homogeneity of particle distribution and improve interfacial bonding, addressing the materials and manufacturing challenges faced in the conventional casting processes [22]. The combination of stirring and squeezing in the two-step process helps minimize porosity by promoting particle dispersion and reducing the likelihood of air entrapment during casting. The even distribution of reinforcement particles and the adequate bonding achieved through squeezing contribute to a refined grain structure [23]. This technique’s ability to harness these benefits makes it a significant advancement in the fabrication of ADC 12-MD reinforced alloy [24].

Existing studies predominantly focus on conventional casting methods and traditional reinforcement materials, often overlooking the potential benefits of incorporating industrial waste such as MD. Furthermore, a comprehensive exploration of the specific advantages of the two-step stir-cum-squeeze casting technique, particularly in conjunction with MD reinforcement, remains limited in the literature. This research aims to bridge these gaps by systematically studying the tribo-mechanical and corrosion properties of ADC 12 alloy composites reinforced with varying concentrations of MD.

By exploiting the unique characteristics of MD and the advantages of squeeze casting, we seek to optimize the manufacturing process and achieve superior mechanical and tribological performance, thereby extending the practical applications of ADC 12-based composites. The primary objectives of this research are twofold: first, to fabricate a range of ADC 12-MD composites with varying MD concentrations using a two-stage squeeze casting approach, and second, to comprehensively evaluate the microstructural, mechanical, tribological, and corrosion properties of these composites. We hypothesize that the incorporation of MD will lead to improvements in hardness, tensile strength, compression strength, wear resistance, and corrosion resistance compared to the as-casted ADC 12 alloy due to refined microstructures, effective load transfer mechanisms, and the potential wear and corrosion barrier effect of MD particles.

The ADC 12 matrix is reinforced using varying wt% (0, 2, 4, 6, 8, and 10 wt%) of MD initially via stir casting as a base method, which then follows squeeze casting and then subjected to various evaluations to understand its mechanical and tribological properties. The present study begins with a description of the manufacturing progress for MD-reinforced ADC 12 MMC, followed by characterization techniques that include radiographic non-destructive technique (NDT) to ensure the acceptable porosity level, followed by microscopic and SEM analysis, mechanical testing that constitutes hardness test, impact test, tensile test, compression test, and tribological and corrosion analysis.

## 2. Materials and Methods

### ADC 12 Alloy

**Figure 1:**
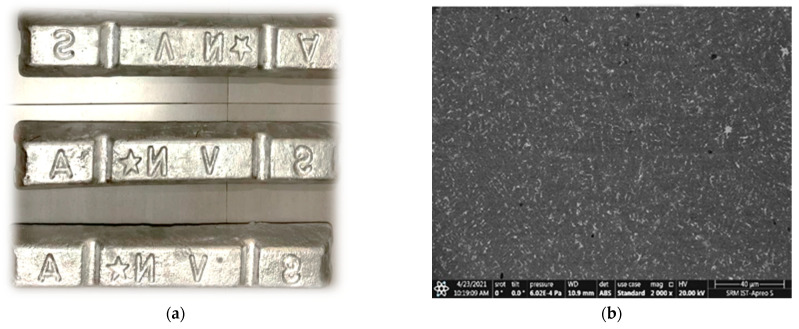
(**a**) ADC 12 alloy billets; (**b**) SEM image of ADC 12 alloy.

Castwell Autoparts Pvt. Ltd. in Chennai supplied the ADC 12 metal for this study. Figure 1a depicts ADC 12 alloy billets, while Figure 1b displays the SEM image of the alloy. As is well-known, ADC 12 is a highly regarded aluminum alloy with several engineering applications, particularly in the automotive industry. The ADC 12 alloy, a widely recognized aluminum–silicon–copper (Al-Si-Cu) alloy, corresponds to the AlSi_9_Cu_3_ alloy according to the DIN EN 1676-2020 standard in Germany and the A380.1 ASTM B179 alloy in the USA. This alloy has applications in various industries, particularly automotive component manufacturing [25]. The alloy primarily consists of aluminum (Al) and silicon (Si) as the major alloying elements, with other trace elements and impurities as listed in Table 1 [26]. Castability, outstanding resistance to corrosion, excellent durability, and promising other mechanical qualities account for the precise properties of these components in the matrix alloy.

The marble dust powder used in this research was sourced from SD Fine Chem—Ltd, Chennai, India. The commercial MD particles are of random size. Therefore, to attain the smaller particles, we sieved it to 300 mesh (i.e., <40 µm), thereby ensuring the particle sizes are smaller, which could aid homogeneous dispersion. The specific gravity of MD is measured to be 2.71 g/cm^3^. Table 2 lists the composition percentages corresponding to MD’s specific chemical components.

**Figure 2:**
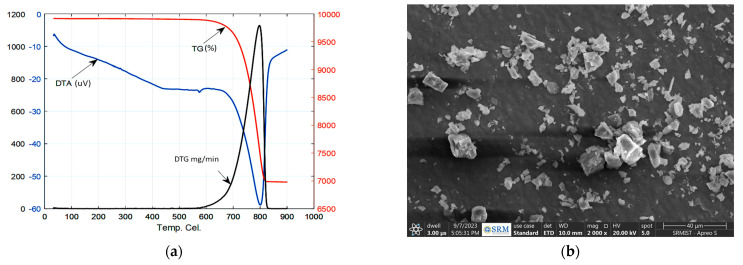
(**a**) DTA/TGA curve for MD; (**b**) SEM image of MD.

Figure 2a,b shows the DTA/TGA curve and SEM image for the MD under consideration, respectively. It can be seen from the TG curve that the MD particles are thermally stable before 620 °C and tend to lose their weight from 620 °C to 820 °C because of the loss of crystal-hydrated water and the Loss of Ignition (LoI). The LoI accounts for volatile components, including water, organic matter, and other trace elements, released as commercial MD undergoes thermal treatment. This loss is attributable to oxidation effects at this temperature range [27].

The DTA/TGA analysis provides valuable insights into the thermal behavior of MD, aiding in optimizing the casting process of the ADC 12-MD composites and identifying the temperature range where significant weight loss and chemical changes occur, making it possible to determine the most suitable temperature for incorporating MD into the ADC 12 matrix, ensuring adequate bonding and uniform distribution of the reinforcement material. This optimization process contributes to achieving enhanced mechanical and tribological properties in the final composite material.

## 3. Production of Composite

**Figure 3:**
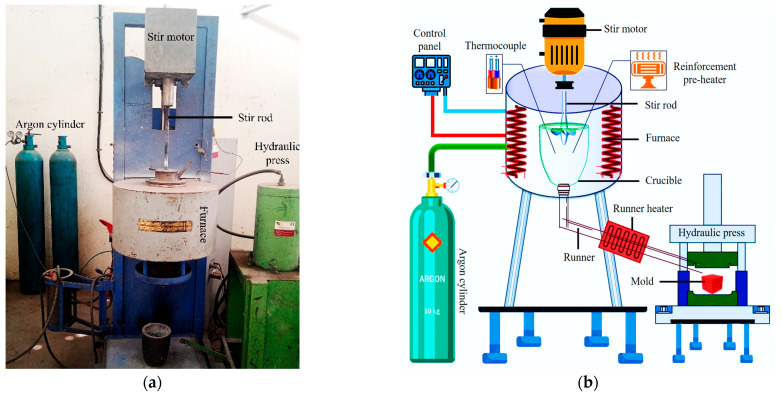
(**a**). Squeeze casting setup; (**b**). Schematic representation of stir-cum-squeeze casting machine.

Squeeze casting was used with a two-stage stir-casting technique to create the composite because it is economical and allows for various material options [28]. The Stir-cum-squeeze casting machine is shown in Figure 3a and is schematically illustrated in Figure 3b. The primary benefit of this approach is that the high-pressure plunger used in anticipation of the molten metal solidifies, eliminates porosity, and ensures equal dispersion of fine particles [29].

The ADC 12 was chopped into billets corresponding to the casting machine’s furnace size to fit into the crucible, which was then melted. In an argon environment, the alloy’s temperature was raised to 740 °C. The molten alloy was dissipated inside this crucible at a temperature ranging from 610 °C to 640 °C when it changed from liquid to semi-solid. After heating for an hour at 300℃, the MD particles were added to all the castings in varying proportions (0, 2, 4, 6, 8, and 10 wt%). Due to the heating process, all remaining moisture was driven out, and the molten ADC 12 alloy wettability was greatly enhanced. Potassium titanium fluoride was added to the melt to improve wettability and interfacial adhesion between the ADC 12 and MD. The wettability of the material was improved further by incorporating 0.1% magnesium powder among the reinforcement and the molten alloy. This resulting slurry was heated to a very high temperature (750 °C) while agitated for 5 to 6 min. The graphite stirrer was laid into the melt for 10 min as a facet of the second stirring phase. The stirrer helped disperse the granules evenly. An external steel mold with a diameter of 50 mm and a length of 300 mm was preheated to 350 °C. The molten composite was then poured into the mold and held for 10 min. The solidification process was facilitated by a hydraulic press applying a pressure of 10 MPa. This high-pressure pressing ensures the equal dispersion of fine particles within the composite material and eliminates porosity. A similar phenomenon was reported in the literature [30]. The composite was introduced into a hot steel mold following processing through a runner connected to the casting apparatus and set to a high temperature. The composite material was fettled from a steel mold at room temperature. Table 3 lists the specimen compositions.

**Figure 4:**
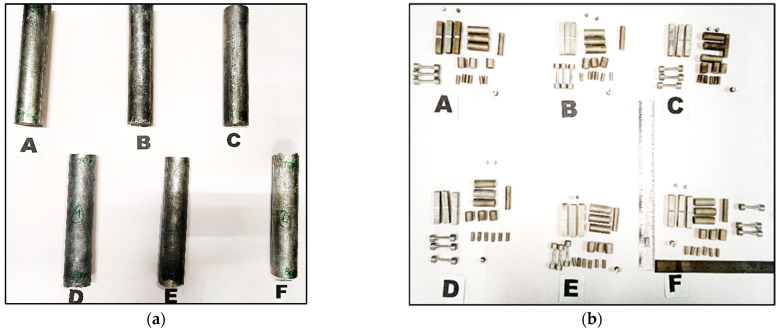
(**a**) Fabricated ingots; (**b**) Specimens wire-cut for characterization studies.

The specimen and ingot were machined using a wire-cut Electrical Discharge Machining (EDM) method to ensure accurate dimensions and prevent unnecessary composite waste. The prepared composite ingots and specimens for testing according to the standards are shown in Figure 4a,b.

## 4. Characterization Studies

### 4.1. Density and Porosity Calculation

Both theoretical and experimental calculations were used to establish the densities of ADC-MD composites. Using the theory of the law of mixtures [31], we can identify the composite’s theoretical density as follows:

$$\rho_{theoretical=\varphi_{m}\rho_{m}+\varphi_{r}\rho_{r}}$$

where $\varphi_{m}$ and $\varphi_{r}$ symbolize the fraction of matrix and reinforcement, respectively, whereas $\rho_{m}$, and $\rho_{r}$, symbolize the corresponding density of matrix and reinforcement. Experimental densities were calculated using an Ohaus density toolbox and Archimedes’ principle [32]. The porosity was reconfirmed using the radiographic non-destructive technique (NDT) for its acceptance level. The quantitative assessment of the distribution of pore sizes in developed composites was made possible by the discrepancy between the theoretical and experimental densities.

### 4.2. Assessment of Mechanical Properties

Tests including tensile, compression, hardness, and impact were conducted to further understand the mechanical characteristics of ADC-MD composites. Computerized universal testing equipment (TUE CN-400/2015/174, 400 kN rating) was used to conduct a tensile test on the material in accordance with ASTM E-8 [33]. The cylindrical samples had an ultimate diameter of 12 mm, a length of 44 mm, a fillet radius of 4 mm, and a gauge measurement of 20 mm to be deployed with wire-cut EDM. The speed of the crosshead turned out to be 1 mm per minute. Following the guidelines of the ASTM E-9 standard [34], a compression test was conducted with the same setup. The specimen measured 13.8 mm × 38 mm (dia × length). The Vickers Hardness tests used the ASTM E-92 standard [32]. The hardness was measured by pressing down on it with a diamond indenter for 10 s at a force of 5 kgf. Impact tests were conducted according to ASTM D-256 [35]. Samples had a size of 10 mm × 10 mm and an angle of 30° at the notch.

### 4.3. Tribological Properties

Pin-on-disc equipment, the standard method for measuring wear rate, is prone to transducer misalignment and measurement precision bias because of the test’s essence [36]. In order to overcome the restriction, this study presents a unique way of employing a linear reciprocating tribometer (LRT) to test the produced samples’ wear as per the ASTM G-133 standard [37]. Microstructural analysis-style preparation involved cutting and polishing specimens to a diameter of 6 × 15 mm (dia × length). Running the tribometer with the specimen safely in place, the stroke length was set to 20 mm, and the frequency was set to 5 Hz/s. Three different load strengths (20 N, 40 N and 60 N) were used in the experiment. Friction was measured as a function of time using the coefficient of friction (CoF).

### 4.4. Corrosion Analysis

The corrosion rate of ADC-MD composites was determined using a continual test that complies with ASTM B-117 standard [38]. Following the samples passed preliminary tests, they were placed in the salt spray chamber and subjected to 72 h of constant spraying with a salt solution (3.5% NaCl) at 35 °C. Deionized water was used to wash the samples before they were dried. The specimens’ weights were recorded before and after the experiment to see any variations. This led us to employ the following equation to determine corrosion rates:

$$Rate\;of\;corrosion\;\left(\frac{mm}{y}\right)=\frac{\left[(87500\times weight\;loss\;(g)\right]}{\left[Density(g/cm^{3}))\times Area(cm^{2})\times exposure\;time(h)\right]}\;$$

### 4.5. Microscopical Analysis

The surface composition and characteristics of ADC-MD composites were studied using field emission SEM equipment (CIQTEK SEM 5000). A computerized interface initiated the device after the produced specimen had been set in the holder. Surface morphology (through SEM images) is also documented.

## 5. Results and Discussion

### 5.1. Effect of MD on Microstructural Analysis

**Figure 5:**
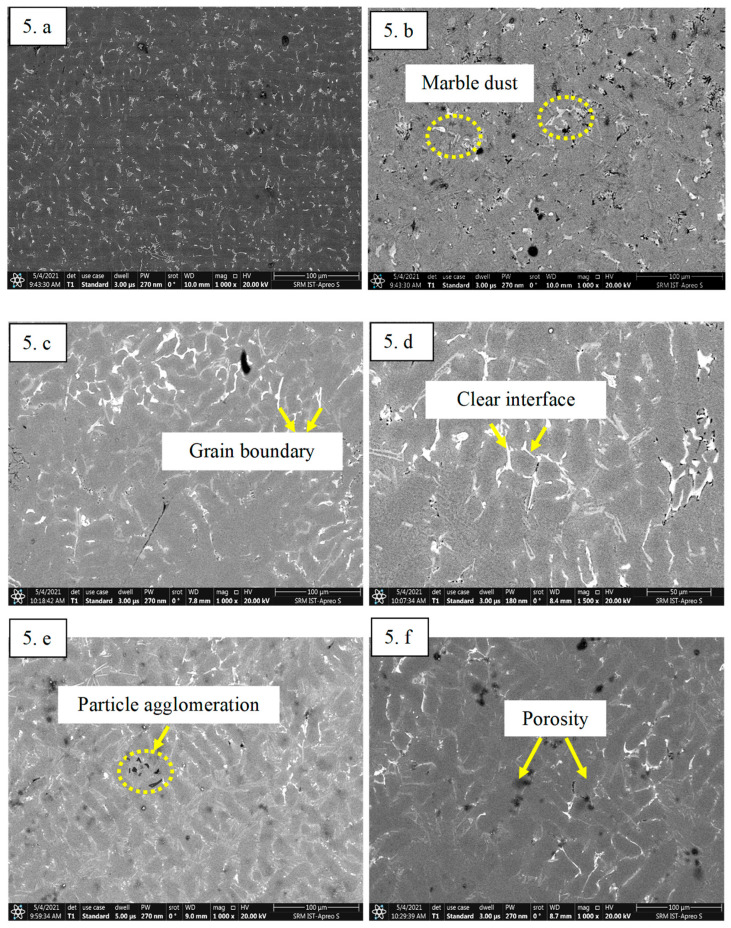
SEM image of (**a**) ADC 12 alloy; (**b**) Squeeze cast ADC 12 with 2 wt% MD; (**c**) with 4 wt% MD; (**d**) with 6 wt% MD; (**e**) with 8 wt% MD; (**f**) with 10 wt% MD.

The microstructure analysis of the composites enables us to gain insights into the distribution and interface between MD particles and the ADC 12 alloy matrix. Figure 5a–f presents the SEM images of the fabricated samples, revealing a homogeneous dispersion of MD particles within the ADC 12 alloy. This uniform distribution is a significant achievement in composite fabrication, as it ensures effective load transfer between the matrix and reinforcements, contributing to improved mechanical properties and overall performance [39]. Moreover, the distinct interface observed between the matrix and MD reinforcement particles indicates a robust interfacial bonding, crucial for load-carrying capability and mechanical efficiency in composite materials. This phenomenon aligns similarly with Mohammed and Chen’s study [40]. The robust interfacial bonding minimizes the possibility of particle debonding or pull-out during mechanical loading, leading to enhanced structural integrity and superior mechanical properties [41]. The homogeneity in particle distribution and the well-bonded interface also play a pivotal role in optimizing the tribological characteristics of the composites, enhancing wear resistance, and reducing frictional losses [41]. In addition, it is vital to acknowledge the size of particles has a profound influence on the hardening effects. The MD particles are in the range of 3–5 µm on average, where such smaller particles often lead to a higher surface area-to-volume ratio, which can enhance the strengthening effect within the material. Such small particles are accountable for the homogeneity in dispersion and are negligible regarding the occurrence of any localized areas of stress concentration; smaller particles may contribute to improved fracture toughness by promoting crack deflection or bridging [42].

### 5.2. Effect of Density and Porosity

**Figure 6:**
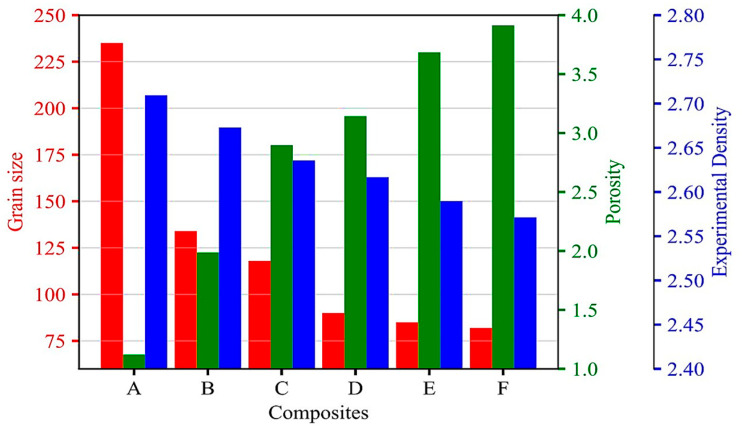
Experimental density and the corresponding grain size and porosity of ADC 12 alloy reinforced with varying wt% of MD.

Figure 6 demonstrates the experimental density, along with the porosity values and measured grain size, for the composites with varying MD wt% (2, 4, 6, 8, and 10 wt%).

It can be seen that the experimental density of the composites decreases as the MD content increases. The experimental density, however, exhibits a more pronounced decrement with increasing MD content, demonstrating a decreasing trend—the percentage difference in porosity increases from 1.24% in A to 3.91% in F. The reduction in experimental density is attributed to the lower density of the MD particles compared to the ADC 12 alloy, leading to increased porosity with higher MD contents. The grain size of the composites also exhibits a decreasing trend with increasing MD content, which is attributed to the finer particle size of the MD reinforcement [43]. The decrease in grain size enhances the interfacial bonding and contributes to improved mechanical properties.

Conversely, the higher MD contents may exhibit higher porosity, primarily due to MD particles’ non-uniform distribution and agglomeration, hindering their efficient packing within the ADC 12 matrix during the casting process. Consequently, the increased porosity adversely affects the mechanical properties, leading to a decrement in experimental density. While lower MD contents (B and C) show a minimal decrease in density and moderate porosity, Composite D demonstrates a balance between reduced density and acceptable porosity. However, at higher MD contents (E and F), the increment in porosity becomes more pronounced, impacting the overall mechanical performance. A similar trend was reported by Kim et al. [44]. Considering the trade-off between density reduction and porosity increase is essential to optimize the MD content for desired mechanical performance. These findings establish the relationship between MD content and the composites’ density, porosity, and grain size, highlighting the significance of optimizing MD content for achieving desirable properties.

### 5.3. Tensile Strength

**Figure 7:**
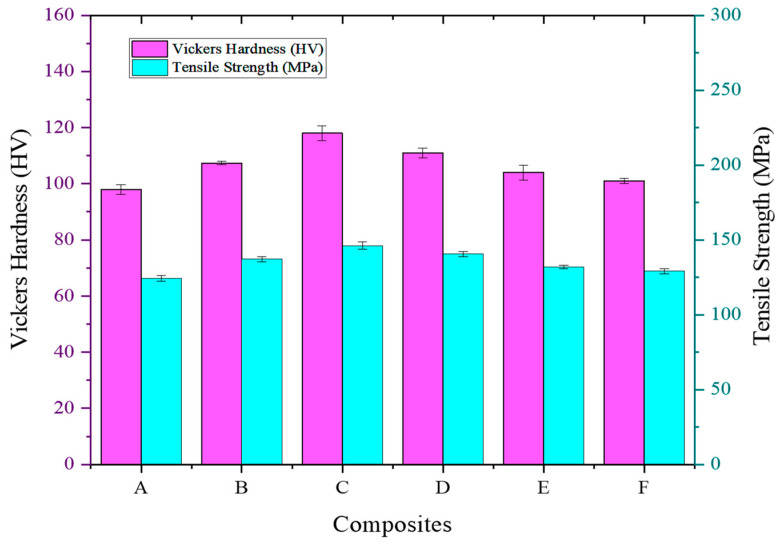
Influence of MD reinforcement on tensile strength of ADC 12 alloy with respect to hardness.

In conjunction with the Vickers Hardness values plotted in Figure 7, the obtained tensile strength measurement results provide crucial insights into the mechanical performance of the ADC 12-MD composites. The Vickers Hardness values (Hv) gradually increase with increasing MD content, indicating improved material hardness.

This hardness enhancement is attributed to the finer grain size and improved interfacial bonding resulting from the incorporation of MD particles. Additionally, the material’s improved resistance to any deformation could be attributed to the finer grain size.

As the MD content increases, grain size refinement leads to stronger interlocking between the matrix and MD particles, contributing to enhanced load transfer mechanisms and improved hardness [45]. The corresponding tensile strength (MPa) measurements demonstrate a similar trend, with a gradual increase observed from A (124.3 MPa) to C (146.2 MPa), which then decreased to F (129.2 MPa). The improved tensile strength is in correlation with the hardness increment, further emphasizing the importance of optimized grain size and interfacial bonding in enhancing the mechanical properties of the composites. Composite C exhibits the highest Vickers Hardness (118 Hv) and tensile strength (146.2 MPa), indicating an optimal MD content resulting in the most favorable microstructure and mechanical performance. However, it is essential to consider the impact of porosity on mechanical properties. Although Composite C shows a relatively high Vickers Hardness and tensile strength, the associated porosity (2.897%) should be considered in the overall assessment of the mechanical performance, which is responsible for the decreased trend in values. High porosity can act as stress concentration points and diminish the mechanical integrity of the material [46]; Composite C, with the optimal MD content, showcases the best hardness and tensile strength combination, indicating the potential for high-performance engineering applications. In addition, both Orowan strengthening and Hall–Petch strengthening mechanisms synergistically contribute to the enhanced hardness and tensile strength observed in the ADC 12-MD composites as well. The refinement of grain size and the presence of MD particles as obstacles to dislocation motion play a pivotal role in optimizing the mechanical performance of the composites [47].

### 5.4. Compression Strength

**Figure 8:**
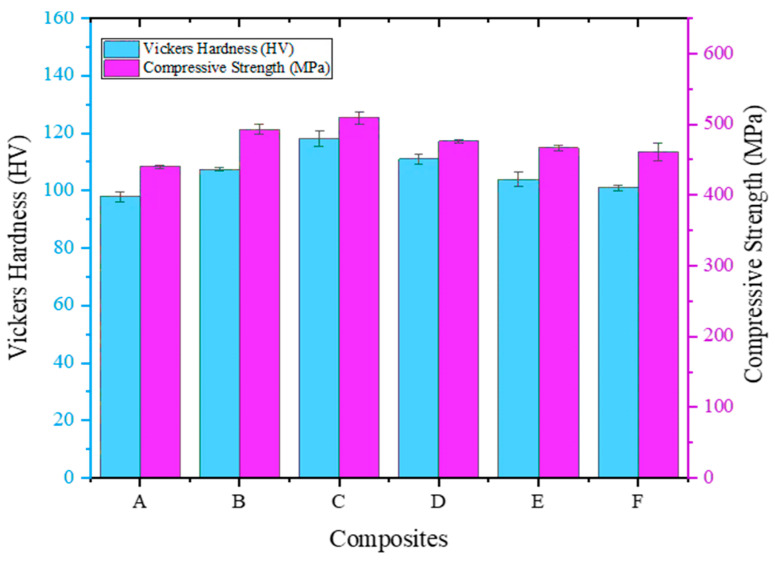
Influence of MD reinforcement on compression strength of ADC 12 alloy with respect to hardness.

The obtained compression strength measurement results and the Vickers Hardness values are given in Figure 8. The Vickers Hardness values gradually increase with increasing MD content, indicating enhanced material hardness. This hardness enhancement can be attributed to the combined effects of Orowan and Hall–Petch strengthening mechanisms. The finer grain structure resulting from the incorporation of MD particles enhances the Hall–Petch strengthening, leading to improved hardness [47].

A similar trend was observed regarding compression strength, where the composite’s compression strength increases from A to F with increasing MD content. The rise in compression strength is in line with the increment in hardness, highlighting the importance of grain size refinement and interfacial bonding in enhancing both tensile and compression properties. The Orowan strengthening mechanism, involving the hindrance of dislocation movement by MD particles, contributes to the increased resistance to plastic deformation during compression testing [48]. Moreover, Composite C exhibits the highest Vickers Hardness and compression strength, reinforcing the correlation between hardness and compression performance in ADC 12-MD composites. The balance between grain refinement and porosity, particularly in Composite C, is crucial in achieving optimal hardness and compression strength.

### 5.5. Impact Energy

**Figure 9:**
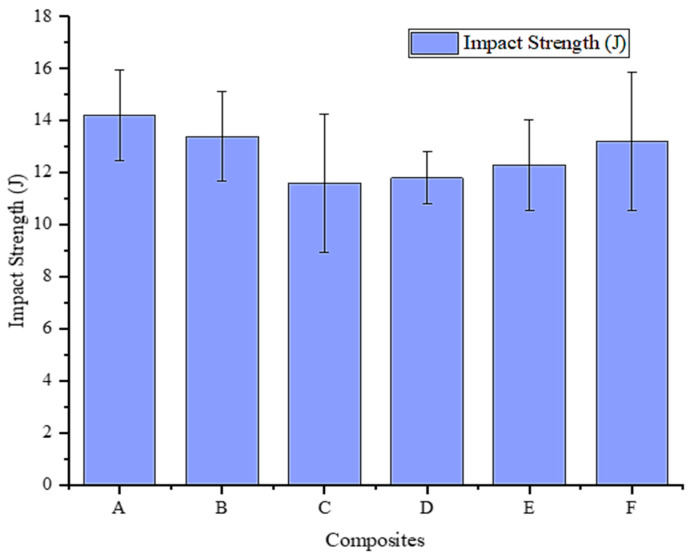
Influence of MD reinforcement on the impact energy of ADC 12 alloy.

Figure 9 displays a plot of the impact energy measurements’ results. The impact energy values indicate the energy absorbed by the composites during the impact test. The impact energy values show a decreasing trend from A to C, followed by a slight increase in D and a gradual rise from E to F.

The observed variations in impact energy can be attributed to the combined effects of the microstructural features, porosity, and MD content. As the MD content increases, the finer grain structure resulting from Hall–Petch strengthening may improve hardness and tensile properties [49], as observed in the previous results. However, it also reduces impact energy, especially in Composite C, where the fine grain structure and increased porosity affect the energy absorption capability during impact loading. Composite D exhibits a slight increase in impact energy compared to C, which may be attributed to a better balance between hardness, tensile strength, and porosity. The impact energy remains relatively higher at lower MD contents (A and B) due to the comparatively coarser grain structure, which can absorb more energy during impact events. As observed in the results, it is essential to consider that porosity can act as stress concentration points and affect the composite’s impact resistance [50]. Composites E and F show a gradual increase in impact energy with a concurrent decrease in porosity, indicating the potential benefits of optimized MD content in achieving enhanced impact properties.

In addition, it is noteworthy that there is an inverse trend in impact energy results in contrast to the hardness, tensile strength, and compression strength of the composites. This could be attributed to potential stress concentrators, such as porosity or insufficient wetting. The porosity and poor wetting can weaken the material and reduce its static mechanical properties such as hardness, tensile, and compression strength [51]. Conversely, they can enhance dynamic properties such as impact energy absorption [52]. Porosity and incomplete wetting create localized weakness within the matrix, allowing for more energy absorption during dynamic impact events as the stress concentrates on these defects, resulting in higher impact energy values [53].

### 5.6. Effect of MD on Wear Rate and CoF of ADC 12 Composite

**Figure 10:**
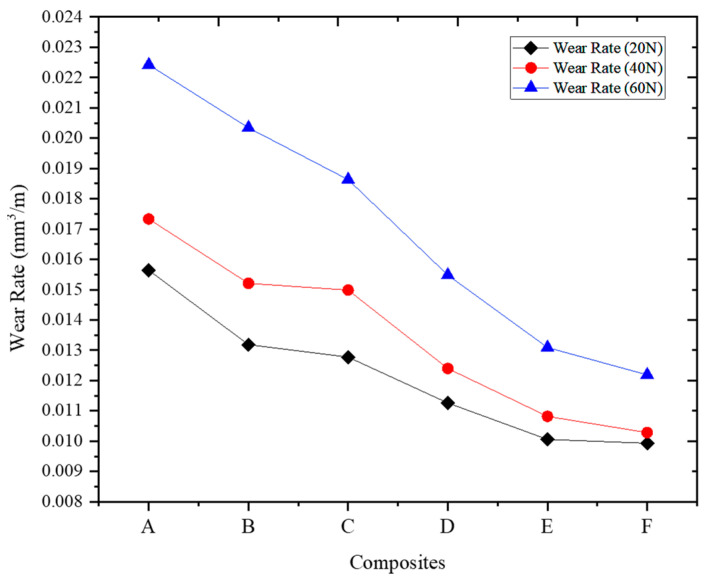
The wear rate of the MD-reinforced ADC 12 composites varies loads.

Figure 10 shows the findings of the wear rate analysis, which provides insights into the wear resistance of the ADC12-MD composites under different applied loads (20 N, 40 N, and 60 N). The wear rate values consistently decrease across all composites as the MD content increases. This indicates that adding MD particles improves wear resistance in the ADC 12-MD composites.

At a load of 20 N, Composite F exhibits the lowest wear rate, followed closely by Composites E and D. As the applied load increases to 40 N and 60 N, the trend remains consistent, with Composite F consistently showing the lowest wear rate, followed by E and D. The wear rate reduction is attributed to the effective dispersion and interlocking of MD particles within the ADC 12 matrix [54], resulting in enhanced load transfer mechanisms and reduced wear. Composite A, which contains no MD reinforcement, exhibits the highest wear rate across all applied loads, indicating the lowest wear resistance among the composites. As the MD content increases from A to F, the wear rate progressively decreases, with Composite F demonstrating the highest wear resistance at all applied loads. The wear rate analysis further reinforces MD reinforcement’s positive impact on the composites’ wear resistance. The refined microstructure resulting from the incorporation of MD particles contributes to reduced wear and frictional losses [55], making the ADC 12-MD composites more suitable for wear-critical applications.

**Figure 11:**
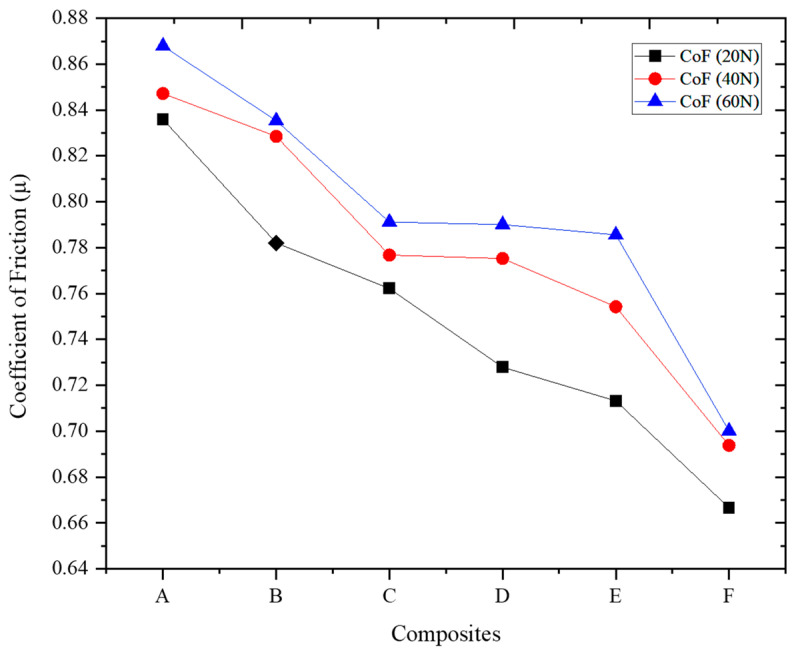
CoF the MD reinforced ADC 12 composites varying loads.

The coefficient of friction (CoF) results to understand the tribological behavior of the ADC 12-MD composites under different applied loads (20, 40, and 60 N) were plotted in Figure 11. The coefficient of friction represents the ratio of the force of friction between two surfaces to the force pressing them together, indicating the level of frictional resistance between the composite and the opposing surface [56]. The CoF values consistently decrease across all composites as the MD content increases. Adding MD particles improves tribological performance in the ADC 12-MD composites [57].

At a load of 20 N, Composite F exhibits the lowest CoF (0.67), followed by Composites E (0.7132) and D (0.7279). As the applied load increases to 40 and 60 N, the trend remains consistent, with Composite F consistently showing the lowest CoF, followed by E and D. The lower CoF values indicate reduced friction and better lubrication properties in these composites due to the incorporation of MD particles [58]. Composite A, which contains no MD reinforcement, exhibits the highest CoF across all applied loads, indicating higher frictional resistance than the other composites. As the MD content increases from A to F, the CoF progressively decreases, with Composite F demonstrating the lowest CoF at all applied loads. The reduced CoF in the ADC 12-MD composites can be attributed to the effective dispersion of MD particles and their ability to act as solid lubricants, reducing the frictional forces between the composite and the opposing surface. The CoF analysis further strengthens MD reinforcement’s positive impact on the composites’ tribological behavior. The improved lubrication properties and reduced friction make the ADC 12-MD composites more suitable for applications where low friction and wear are critical considerations.

### 5.7. Effect of MD on Corrosion Behavior of ADC 12 Composite

**Figure 12:**
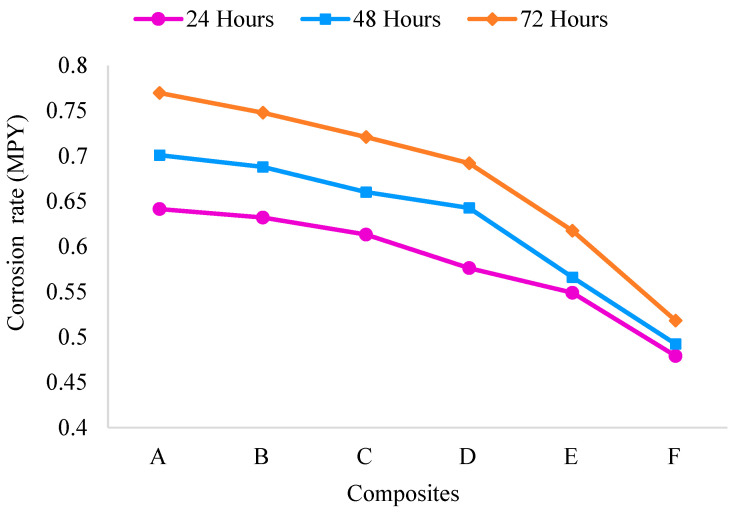
Corrosion behavior of MD reinforced ADC 12 composite.

The corrosion results for the ADC 12-MD composites were obtained by measuring the corrosion rate over different exposure periods of 24 h, 48 h, and 72 h and are plotted in Figure 12.

The corrosion rate decreases as the exposure time increases for all composites. This indicates that the corrosion resistance of the ADC 12-MD composites improves with more prolonged exposure to the corrosive environment. Composite F exhibits the lowest corrosion rate among the composites, followed by E, D, C, B, and A at all three exposure periods. As the MD content increases from A to F, the corrosion rate progressively decreases, emphasizing the beneficial effects of MD reinforcement on enhancing the corrosion resistance of the composites. The improved corrosion resistance can be attributed to the MD particles’ uniform distribution and effective barrier properties, which inhibit corrosive agents’ penetration into the composite matrix. MD particles act as a corrosion inhibitor, protecting the ADC 12 matrix from corrosive attack [59].

## 6. Conclusions

The incorporation of marble dust (MD) as a reinforcement material in ADC 12 aluminum alloy composites to enhance their tribo-mechanical and corrosion resistance properties was investigated to explore an alternative and sustainable approach to overcome the limitations of conventional ceramic reinforcements and casting techniques. The composites were fabricated for varying MD proportions (2, 4, 6, 8, and 10 wt%) via a two-step stir-cum-squeeze casting approach, facilitating uniform particle dispersion and improved interfacial bonding. An extensive evaluation of various mechanical and tribological properties found that Composite C, with 4 wt% MD, emerged as the optimal composite. Composite C demonstrated significant improvements over Composite A (as casted ADC 12) in various aspects as follows:

- Compared to Composite A, Composite shows an increase in hardness, tensile, and compression strength by 20.41%, 17.63%, and 15.75%, respectively. Incorporating MD particles contributed to grain size refinement, improving the composites’ hardness, tensile strength, and compression strength. Orowan strengthening and Hall–Petch strengthening mechanisms synergistically contributed to the enhanced mechanical properties observed in the ADC 12-MD composites.
- Furthermore, the Composite C exhibited a lower wear rate and CoF by 18.33% and 8.8%, respectively. The uniform distribution and effective interlocking of MD particles within the ADC 12 matrix contributed to reduced wear and frictional losses.
- Moreover, the corrosion rate of the Composite C was 4.39%, 4.44%, and 17.57% lower than the as-casted ADC 12 alloy, tested at 24 h, 48 h, and 72 h, respectively, showcasing enhanced corrosion resistance over time. MD particles acted as corrosion inhibitors, protecting the ADC 12 matrix from corrosive attack.

Overall, the successful incorporation of MD as a reinforcement material addressed the challenges associated with conventional ceramic reinforcements and aligned with sustainable practices by utilizing industrial waste. Composite C, with 4 wt% MD, offers promising potential for high-performance engineering applications while contributing to responsible consumption and production (SDG 12).

Future research could explore a broader range of MD percentages to identify the most suitable compositions for specific applications. Real-world testing and application-specific assessments are necessary to evaluate the composites’ performance under specific operating conditions. Furthermore, exploring the influence of processing parameters and manufacturing techniques on the composite properties could further enhance fabrication processes and overall material performance. By continuously refining our understanding of MD-reinforced ADC 12 composites, we can unlock their full potential for a wide range of engineering applications.

## Figures and Tables

**Table 1:** The elemental composition (in mass fraction) of the ADC 12 matrix alloy.

Si	Cu	Mg	Zn	Fe	Mn	Ni	Sn	Al
10.6%	2%	0.3%	1%	1.3%	0.5%	0.5%	0.3%	Remain

**Table 2:** Chemical composition of MD.

Chemical Component	Composition Content in %
SO_3_	0.02
K_2_O	0.91
Na_2_O	0.63
MgO	18.94
CaO	32.23
Fe_2_O_3_	1.09
Al_2_O_3_	1.09
SiO_2_	4.99
Loss of Ignition (LoI)	40.1%

**Table 3:** The constituent elements of a composite specimen.

Label	ADC 12	MD (wt%)
A	100 wt%	0 wt%
B	98 wt%	2 wt%
C	96 wt%	4 wt%
D	94 wt%	6 wt%
E	92 wt%	8 wt%
F	90 wt%	10 wt%

## Data Availability

The data are reported within the article.
